# Prevalence of Metabolic Syndrome and Its Components in Bamboutos Division's Adults, West Region of Cameroon

**DOI:** 10.1155/2019/9676984

**Published:** 2019-04-30

**Authors:** Wiliane J. T. Marbou, Victor Kuete

**Affiliations:** Department of Biochemistry, Faculty of Science, University of Dschang, Dschang, Cameroon

## Abstract

The prevalence of metabolic syndrome (MetS) and its associated risks remain unappreciated in Bamboutos Division, west region of Cameroon. This study aimed to evaluate the prevalence of MetS, its individual components, and associated risk factors among Bamboutos Division's adults population using a Joint Interim Statement of the International Diabetes Federation (IDF) Task Force on Epidemiology and Prevention definitions parameters. A cross-sectional study was conducted from May 2016 to May 2018 in Mbouda ADLUCEM Hospital and Mbouda District Hospital, two reference hospitals in Bamboutos Division, west region of Cameroon. Interview, physical and clinical examinations, and lipid and fasting blood glucose measurements were conducted for 604 adults. The definition of MetS proposed by IDF was used. The prevalence of MetS was 32.45% with highly significant female predominance (46.11% for females and 14.01 % for males). In the entire participants, the most common abnormalities were low-HDL (82.78%) and hypertriglyceridemia (53.97%) [p<0.001]. Participants with obesity (OR: 16.34; 95% CI: 9.21-28.96), overweight (OR: 7.45; 95% CI: 4.17-13.30), and highest hs-CRP (hs-CRP >11 mg/l) had a higher risk of developing MetS. The most common MetS component was abdominal obesity (OR: 353.13; 95% CI: 136.16-915.81). MetS is prevalent among Bamboutos Division's adults in west region of Cameroon and abdominal obesity is the most common MetS component. This study highlights the need for evidence-based prevention, diagnosis, and management of MetS and its associated factors among Bamboutos Division's adults in Cameroon.

## 1. Introduction

Noncommunicable diseases are increasingly important causes of morbidity and mortality in Africa due to the rapid demographic transition and changing in lifestyle. In African countries, the prevalence of diabetes in 2017 was 3.3%, the overall prevalence of hypertension was 55.2% in 2017, and overweight and obesity were estimated to cause 3.4 million deaths in 2017 [1–3]. Concern about the health risks associated with rising noncommunicable diseases has become nearly universal in Africans countries. Cardiovascular diseases and type 2 diabetes are recognized as a determining factor in the development of metabolic syndrome (MetS) [4, 5]. Metabolic disorders known as the “metabolic syndrome” are defined as a set of biological and anthropomorphic disturbances whose most easily observable clinical marker is overweight, especially abdominal obesity [4]. According to the available data, foodstuffs rich in fatty and/or sugary foods, combined with low energy outgo, constitute one of the main aetiologies of the MetS. The prevalence of the MetS varies between different populations in Africa. This prevalence also varies according to the criteria used to define MetS, the type of population recruited, and the type and age of the subjects. In addition, studies show an increase in prevalence over time, as a result of changes in diets and lifestyle in developing countries [6]. Thus, the MetS is an emerging public health problem in developing countries.

The prevalence of MetS in Africa ranges from 0% to 50% or even higher depending on the population and the criteria used in defining it [7]. The prevalence of MetS was found to be 35.1% (ATP III) in northwestern Nigeria, 21.8% (IDF) in adults in South Africa attending healthcare facilities in Eastern Cape, 35.73% (IDF) among adults in Morocco, 25.6% among urban Kenyan population, and 38.98% (IDF) in adult men in the Dschang Health District in the west region of Cameroon [8–12]. In Cameroon, studies on the distribution of the individual components of MetS are very limited. This study aimed to evaluate the prevalence of MetS, its individual components, and associated risk factors among Bamboutos Division's adults as defined by the Joint Interim Statement of the International Diabetes Federation Task Force on Epidemiology and Prevention [13].

## 2. Materials and Methods

### 2.1. Study Type and Population

**Figure 1:**
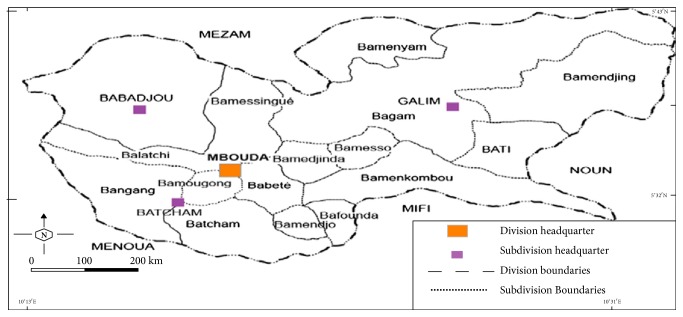
Map of Bamboutos Division with subdivision headquarters in west region of Cameroon [14].

This was a cross-sectional study conducted from May 2016 to May 2018 in Mbouda ADLUCEM Hospital and District Hospital, two reference hospitals in Bamboutos Division, west region of Cameroon (Figure 1). Participants were randomly selected and included in the study if they were 20 years old and older, attending the two reference hospitals. The human immunodeficiency virus- (HIV-) positive patients, pregnant women, and participants with positive serology for hepatitis B and C were not included in this study.

### 2.2. Sociodemographic and Anthropometric Data Collection

A structured questionnaire was used to gather information on sociodemographic variables (gender, age, and level of education), smoking status, and physical activity. Smoking was categorized as current, former, and never. Former smokers were those who reported that they had smoked cigarettes during their lifetime but are not currently smoking cigarettes. Drinking was categorized as never, moderate, and heavy. Never drinkers were those who did not drink beer, wine, or hard liquor during the past month. Moderate drinkers had an alcoholic beverage (beer, wine, or hard liquor) less than once per day during the past month. Heavy drinkers were defined as those who ever drank 4 or more alcoholic beverages per day or who drank beer, wine, or hard liquor 1 time per day during the past month. Physical activity was categorized as low (walking ≤ 60 min/day), moderate (walking ≥ 60 min/day), high (vigorous activity for ≤ 30 min/day), and very high (vigorous activity for ≥ 30 min/day) physical activities.

Trained staff measured anthropometric measurements including weight and height. Body mass index (BMI) was tricked out by dividing the weight in kilograms by the square of the height in meters. BMI was categorized as underweight, <18.5 kg/m^2^, normal, 18.5-24.9 kg/m^2^, overweight, 25-29.9 kg/m^2^, and obesity, ≥30 kg/m^2^ [15]. Blood pressure (BP) was measured using standardized sphygmomanometer. Trained personnel performed the procedures while the subject was in a sitting position with the arm at the level of the heart and after 5 minutes' rest. Two blood pressure readings were taken from each participant and the average reading of both was used in this study. The participant was labelled as having hypertension if systolic blood pressure was ≥ 130 mm Hg or diastolic blood pressure was ≥ 85 mm Hg or if the patient was on antihypertensive medications [13]. At the level of iliac crest, precisely at minimal respiration to the nearest 0.1 cm, the waist circumference was measured.

### 2.3. Laboratory Measurements

Venous blood was collected after overnight fasting to determine fasting plasma glucose (FPG) using Accu-Chek® Active reader, as described by the manufacturer (Roche Diabetes Care GmbH, Sandhofer Strasse 116, 68305 Mannheim, Germany). Plasma concentrations of total cholesterol and triglycerides (TG) were evaluated by enzymatic methods. High-density lipoprotein (HDL) cholesterol was evaluated by enzymatic colorimetric method. Low-density lipoprotein (LDL) cholesterol was calculated using the Friedewald formula if the triglycerides are less than 400 mg/dl (4.6 mmol/l) [16]. Biochemical tests were performed using a MaestroNano® Pro Spectrophotometer (Maestrogen, 8275 South Eastern, Avenue #200, Las Vegas, NV 89123, USA) and the reference commercial kits (Sigma-Aldrich Co., 3050 Spruce Street, St. Louis, MO 63103, USA). We analyzed high sensitivity C-reactive protein (hs-CRP) using ELISA solid phase direct sandwich method (Sigma-Aldrich, St. Louis, USA) with ELx808™ Microplate reader (BioTek Instruments, Winooski, USA).

### 2.4. Definitions of Metabolic Syndrome and Dyslipidemia

The criteria used for the definition of metabolic syndrome in adults specified by IDF were applied. Therefore, subjects were considered to have MetS if they had central obesity that was defined by a waist circumference ≥ 94 cm in men and ≥ 80 cm in women, along with two or more of the following criteria, as per the Joint Interim Statement of the International Diabetes Federation Task Force on Epidemiology and Prevention [13]: high fasting glucose level ≥ 100 mg/dL (5.6 mmol/L) or patients known to have diabetes mellitus and/or on treatment for diabetes; hypertriglyceridemia-serum triglyceride level ≥ 150 mg/dL (1.7 mmol/L); low HDL cholesterol-serum; HDL cholesterol < 40 mg/dL (1.0 mmol/L) in men and < 50 mg/dL (1.3 mmol/L) in women or patients known to have dyslipidemia; high blood pressure [systolic blood pressure (SBP) ≥ 130 mmHg and/or diastolic blood pressure (DBP) ≥ 85 mmHg or patients known to have hypertension and/or on treatment for hypertension]. Dyslipidemia was defined as specified by American Association of Clinical Endocrinologists and American College of Endocrinology [17].

### 2.5. Assessment of Dietary Intake Frequency

In the present study, dietary intake over the past month was assessed with a structure questionnaire. The questionnaire includes 23 food items highly consumed in the area. Participants were asked about the frequency of each food consumed during the month of their participation in the study. According to the frequency of food intake, each food item was classified into four intervals ([0-4], [4-13], [13-25], and [25-90] times/month).

### 2.6. Ethical Consideration

Ethical approval was obtained from the Cameroon National Ethics Committee (CNEC), Ministry of Public Health (reference number, 2018/06/1054/CE/CNERSH). Prior to data collection, permission was obtained from each manager of the Mbouda ADLUCEM and District Hospital. Information sheets detailing the purpose and process of the study were provided to each participant. Each participant gave written, informed consent for his/her voluntary participation.

### 2.7. Statistical Analysis

All statistical tests were two-tailed and statistical significance was set at p<0.05. Continuous variables were expressed as mean values ± Standard Deviation (SD) and compared by using Welch* t*-test. Categorical data were expressed as frequency and compared by using Chi-square tests. We calculated the prevalence of MetS according to the IDF criteria. Logistic Regression Model was used to evaluate the association between MetS and associated risk factors. Epi Info™ version 7.2.2.6 (CDC, 1600 Clifton Road, Atlanta, GA 30329-4027, USA) was used for statistical analyses.

## 3. Results

The features of Mbouda's adults by sex who participated in this study are presented in Table 1. 604 [57.45% (n=257) males and 42.55% (n=347) females] participated in this study with the average age of 43.74±17.21 years. There was a significant difference between the two groups in terms of educational level, drinking and smoking status, physical activities, abdominal obesity (resp., p<0.001), and serum hs-CRP (p=0.008). Female participants (45.87±16.87 years) were more likely than male participants (40.87±17.29 years) to be older, with lower levels of systolic blood pressure (Table 1).

The features of the participants according to MetS status are presented in Table 2. Participants with MetS (48.19±15.48 years) were more likely (p<0.001) than the normal participants (41.60±17.61 years) to be older, with higher (p<0.001) levels of total cholesterol, glycaemia, DBP, waist circumference, BMI, and serum hs-CRP (resp., 175.75±85.83 mg/dl; 110.14±49.85 mg/dl; 83.04±12.94 mmHg; 96.56±14.40 cm; 31.56±6.46 Kg/m^2^; 13.72±26.96 mg/l for participant with MetS compared to 152.25±70.01 mg/dl; 93.50±22.38 mg/dl; 79.11±12.17 mmHg; 77.50±13.89 cm; 25.48±4.91 Kg/m^2^; 2.12±3.94 mg/l for normal participants). MetS participants were more likely to have abdominal obesity (95.41%), obesity (56.12%), diabetes (44.90%), and hypertension (58.16%) (Table 2).

**Figure 2:**
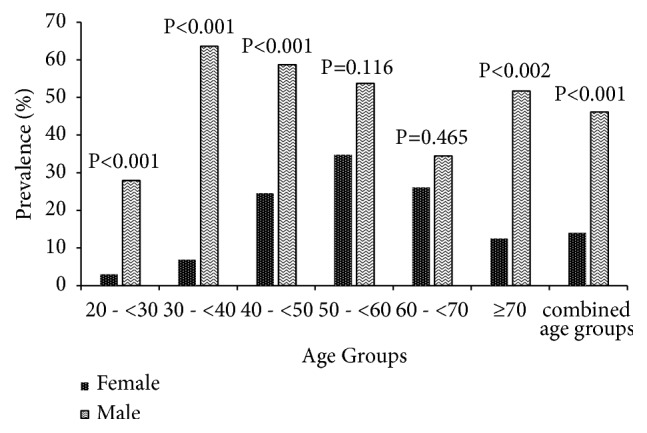
Variation in the prevalence of metabolic syndrome by sex and age group in the entire population (n=604). P value (between males and females); MetS, metabolic syndrome.

The variation in the prevalence of MetS by sex and age in the study participants is shown in Figure 2. The overall prevalence of MetS was 32.45 % with highly significant females predominance (46.11% for females and 14.01 % for males; p<0.001). The data show an increase of MetS prevalence with age up to 59 years. A small decline in this prevalence was observed in patients aged 60 years and above (Figure 2). The highest prevalence of MetS was found in male (34.78%) participants aged 50-<60 years and female (63.64 %) participants aged 30-<40 years [also see Table S1].

**Figure 3:**
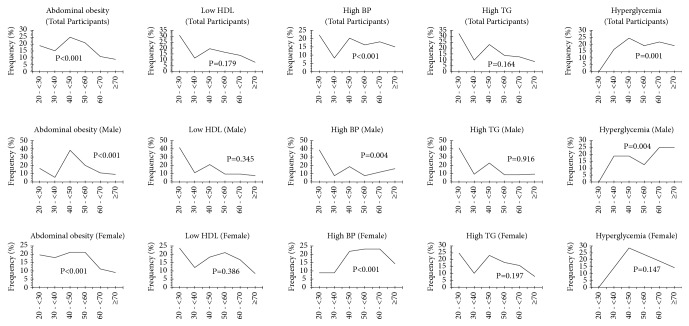
Frequency (%) of MetS components by sex and age groups in the study population. P value (between age groups); MetS, metabolic syndrome; HDL, high-density lipoprotein; n, size; BP, blood pressure.

The variation in the frequency of risk factors associated with MetS by sex and age group in the population is presented in Figure 3. According to these results, in the entire participants, the most common abnormalities were low-HDL (82.78%) and hypertriglyceridemia (53.97%). The most affected groups were 20-<30 years (HDL, 30.80%, and hypertriglyceridemia, 32.21%) and 40-<50 years (HDL, 19.40%, and hypertriglyceridemia, 23.01%). In the male and female participants, low-HDL was the common abnormality affecting participants aged 20-<30 years. Figure 3 also shows that abdominal obesity significantly decreases with age, with a maximum among participants aged 40-<50 years in the total, male, and female participants. The majority of abnormalities associated with MetS were expressed in their highest frequencies in participants aged 20-<30 years for some parameters and 40-<50 years for others [also see Table S2].

**Figure 4:**
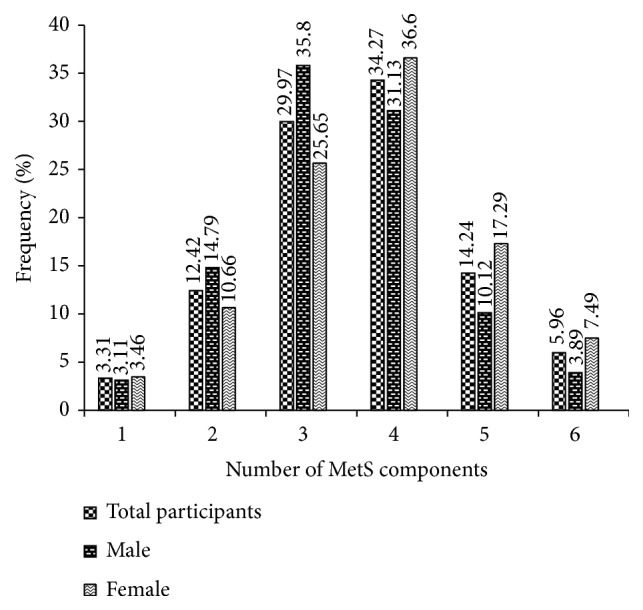
Frequency (%) of the number of metabolic syndrome components by sex in the study population. MetS, metabolic syndrome; 0: no metabolic syndrome components; 1: one metabolic syndrome component; 2: two metabolic syndrome components; 3: three metabolic syndrome components; 4: four metabolic syndrome components; 5: five metabolic syndrome components.

**Figure 5:**
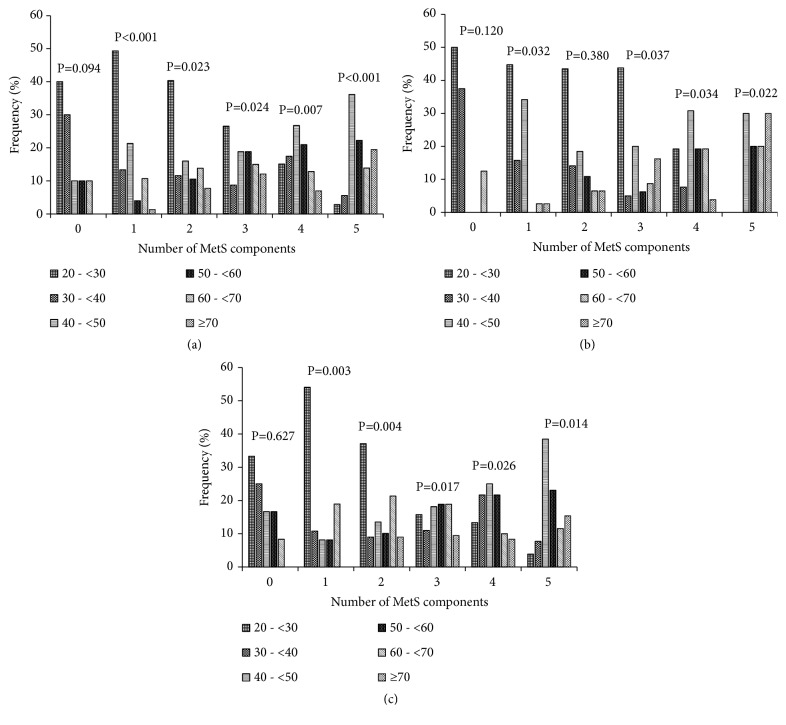
Frequency (%) of the number of metabolic syndrome components by age group in the study population. P value (between age groups); (a) overall population; (b) female participants; (c) male participants; MetS, metabolic syndrome; 0: no metabolic syndrome components; 1: one metabolic syndrome component; 2: two metabolic syndrome components; 3: three metabolic syndrome components; 4: four metabolic syndrome components; 5: five metabolic syndrome components.

In this study, we examined the frequency of the number of MetS components by sex (Figure 4) and age group (Figure 5). In our study population, 34.27% (31.13% in males and 36.60 % in females) of the participants had at least three MetS components, which is the definition of MetS (Figure 4). Those with two risk factors represent 29.97% of the population and are at risk of developing MetS. The participants aged 20-<30 years and 40-<50 years accumulated more risk factors compared to the other age groups among both males and females. In total participants, the variation of severe MetS (five associated anomalies) was significant in all age groups (p<0.001) (Figure 5(a)) [see Table S3 for more details].

The association between sociodemographic parameters, hs-CRP levels, and MetS in total participants was studied and presented in Table 3. Participants with obesity (OR: 16.34; 95% CI: 9.21-28.96) and overweight (OR: 7.45; 95% CI: 4.17-13.30) had a higher risk of developing MetS. Participants aged 50-<60 years (OR: 5.66; 95% CI: 3.17-10.12) had a higher MetS risk (Table 3). This study has shown that participants with the highest hs-CRP (hs-CRP >11 mg/l) had a 4.37-fold increased risk of MetS compared to those with the lowest hs-CRP (hs-CRP [0-11] mg/l).

Logistic regression analysis was used to study the most common MetS definition parameters among the participants (Table 4). According to the results, in the entire population, abdominal obesity (OR: 353.13; 95% CI: 136.16-915.81), low-HDL (OR: 9.28; 95% CI: 3.98-21.62), high-TG (OR: 5.62; 95% CI: 2.69-11.74), high blood pressure (OR: 4.43; 95% CI: 2.27-8.63), and hyperglycemia (OR: 4.24; 95% CI: 2.11-8.52) are the most common abnormalities affecting participants. The results suggest that abdominal obesity is the strongest predictor of MetS in our study participants (Table 4).

Table S4 shows the average frequency of food intake of the participants according to metabolic syndrome. MetS participants consumed more fishes (p<0.001), fufu corn (p=0.010), and cabbage (p=0.045) than normal participants who in turn consumed more pasta (p<0.001) and sugar products (p=0.027) than MetS participants.

Associations between the frequency of foods intake and metabolic syndrome prevalence in total participants are shown in Table S5. A high frequency of fish intake was associated with lower odds of having MetS after adjustment for confounding factors.

## 4. Discussion

In the present study, we evaluated the prevalence of MetS, its individual components, and associated risk factors among Bamboutos Division's adult population using a Joint Interim Statement of the International Diabetes Federation Task Force on Epidemiology and Prevention definitions parameters [13]. Results on features of persons who participated in the study according to sex showed a significant difference between the two groups in terms of educational level, drinking and smoking status, physical activities, abdominal obesity, and serum hs-CRP. Female participants (45.87±16.87 years) were more likely than males participants (40.87±17.29 years) to be older, with lower levels of SBP. Sociodemographic characteristics of population vary geographically and depend on the socioeconomic and sociopolitical development of each region. The sex differences on serum hs-CRP level observed in this study could be explained by endogenous synthesis of hormone like oestrogen that might play a role in the inflammatory process in women. This could also be explained by the fact that women compared to men have a large amount of adipose tissue source of proinflammatory cytokine [18].

According to the MetS status, participants with MetS were more likely older than the normal participants. These results are in agreement with those obtained by Li and collaborators among adults in China [19]. Participants with metabolic syndrome had higher levels of total cholesterol, glycaemia, DBP, waist circumference, BMI, and serum hs-CRP and were more likely to have abdominal obesity (95.41%), obesity (56.12%), diabetes (44.90%), and hypertension (58.16%) compared to normal participants. Previous studies revealed that metabolic dysfunctions such as high blood pressure, hyperglycemia, and obesity are the factors related to the MetS [20–22].

The overall prevalence of MetS in this study was 32.45 % with highly significant female predominance (46.11% for females and 14.01 % for males). The overall metabolic prevalence obtained in this study is similar to the prevalence rate of Dandji et al. (38.98%) among adult men of Dschang Health District [12], Lee et al. (30.52%) in south Korea [4], Brini et al. (35.73%) in Morocco [10], and Sabir et al. (35.1%) in northwestern Nigeria [23]. However, it is high compared to the prevalence in the Eastern Cape, South Africa (21.8%) by Owolabi et al. [9]. High prevalence of the MetS in Mbouda adults may be caused by high prevalence of obesity, hypertension, and diabetes in this population. This may also be due to the lifestyle and some genetics factors. High prevalence of the MetS in females compared to males corroborates with the prevalence rate of Brini et al. (40.12% among women and 18.56% among men) in Morocco [10]. This finding is consistent with that of Belfki et al. in Tunisia [24] and differs with the finding of Santos et al. among South European population [25]. These results might be due to different cut-off points set as criteria for metabolic syndrome like abdominal obesity, low-HDL cholesterol, and hypertriglyceridemia. Women in the menopausal state had a decline in circulating oestrogen levels. This decrease in oestrogen concentration may increase cardiovascular diseases in women through effects on adiposity, lipid metabolism, and prothrombotic state [26]. This is consistent with other results of this study, which have shown that female participants were more likely to be older than male participants.

The study of the variation in the frequency of the MetS components by sex and age group has shown that low HDL (82.78%) and hypertriglyceridemia (53.97%) were the most common abnormalities in the entire participants. It has also revealed that participants aged 20-<30 years and 40-<50 years were the most affected and that abdominal obesity significantly decreases with age with a maximum among participants aged 40-<50 years in the total, male, and female participants. Cameroon's population is currently very young, and the above results are mainly driven by the constant rise in diabetes, obesity, and hypertension in Cameroonian population [27–29]. Diabetes and obesity increase the risk of adipose tissue insulin resistance, which plays important role in the pathophysiology of the MetS [30]. High prevalence of MetS in females throughout the age groups could be explained by the high prevalence of obesity among female participants in this study. It may be also attributable to the steep increase in blood pressure in women after menopause, which initiates a more rapid decrease in endothelial function [31].

Regarding the trend curves of abdominal obesity and hyperglycemia as a function of age groups in males in Figure 3, abdominal obesity would be due to waist circumference values. Waist circumference tended to be higher in younger adults than in older men [32, 33]. A hormonal process involving endogenous oestrogen, which would provoke hyperglycemia in men, could explain the trend curves of hyperglycemia as a function of age groups in males [34].

This study reveals that 29.97% of the population are at risk of developing MetS. Other studies showed that MetS is emerging alarmingly in low-income countries [35, 36]. This may be due to increasing urbanization, westernization of lifestyle including unhealthy diet, physical inactivity, and lack of awareness about metabolic syndrome.

The results on the study of associations between MetS and sociodemographic parameters showed that obesity (OR: 16.34; 95% CI: 9.21-28.96), overweight (OR: 7.45; 95% CI: 4.17-13.30), and participants aged 50-<60 years (OR: 5.66; 95% CI: 3.17-10.12) had a higher MetS risk. They also show that participants with the highest hs-CRP (hs-CRP >11 mg/l) had a 4.37-fold increased risk of MetS compared to those with the lowest hs-CRP (hs-CRP [0-11], mg/l). Considering the ever-increasing body of evidence regarding MetS, chronic low-grade inflammation may have an important role in the pathogenesis of metabolic disorders [37]. Concerning parameters used for MetS definition, abdominal obesity (OR: 353.13; 95% CI: 136.16-915.81), low HDL (OR: 9.28; 95% CI: 3.98-21.62), high-TG (OR: 5.62; 95% CI: 2.69-11.74), high blood pressure (OR: 4.43; 95% CI: 2.27-8.63), and hyperglycemia (OR: 4.24; 95% CI: 2.11-8.52) are the most common abnormalities affecting participants. Obesity is known as a risk factor of the MetS and our study results are consistent with those of Brini et al. [10], Moreira et al. [38], and Carnethon et al. [39]. The results suggest that abdominal obesity is the strongest predictor of MetS in our study participants.

The present study also suggested that the frequent consumption of fishes was associated with lower odds of having MetS. The possible explanation for this association is that fish proteins are easily digestible, rich in essential amino acids, and have been seen to slow absorption and synthesis of lipids and promote the lipid excretion [40].

The main strength of the current study is that it is one of the large-sample-size studies regarding MetS in Cameroon. The results of this study would certainly contribute to the sensitization and the prevention of the MetS in Bamboutos Division. However, several limitations should be considered. First, the cross-sectional design limits the ability to address causal relationships between risk factors and metabolic syndrome. Second, the prevalence of metabolic syndrome was based on a single assessment of blood samples, which may lead to minor inaccuracies. Third, because the sociodemographic characteristics and dietary information were obtained through a questionnaire, this may lead to recall bias. Fourth, the species of fish, preparation methods, seasonal variation, and possible contaminants of fish consumed could not be examined in the current study and assessment of these factors will provide additional information regarding fish-MetS associations.

## 5. Conclusion

The present study discloses high prevalence of metabolic syndrome among our study population and significant females predominance compared to males. In addition, one-third of the study population were at risk of metabolic syndrome. Low-HDL cholesterol, hypertriglyceridemia, high blood pressure, and abdominal obesity were the common abnormalities among participants. Our results suggest specific association between risk factors including sociodemographic features abdominal obesity, low-HDL cholesterol, high blood pressure, and hyperglycemia with metabolic syndrome. The results also suggest that abdominal obesity is the strongest predictor of metabolic syndrome in our study participants. The findings highlight the need for evidence-based prevention, diagnosis, and management of metabolic syndrome and its associated factors among Bamboutos Division adults in Cameroun.

## Figures and Tables

**Table 1:** Features of persons who participated in the study according to sex in total participants.

		Total participants	Males	Females	*p value* (between male and female)
Total, n (%)		604	257 (57.45)	347 (42.55)	
Age (years)^a^		43.74±17.21	40.87±17.29	45.87±16.87	< 0.001

Educational level, n (%)^b^	Uneducated	11 (1.82)	1 (2.88)	10 (0.39)	< 0.001
	Primary	200 (33.11)	66 (38.62)	134 (25.68)	
	secondary	305 (50.50)	136 (48.70)	169 (52.92)	
	University	88 (14.57)	54 (9.80)	34 (21.01)	

Residence area, n (%)^b^					0.487
	Mbouda	424 (70.20)	188 (73.15)	236 (68.01)	
	Batcham	118 (19.54)	45 (17.51)	73 (21.04)	
	Galim	35 (5.79)	15 (5.84)	20 (5.76)	
	Babadjou	27 (4.47)	9 (3.50)	18 (5.19)	

Drinking, n (%)^b^					< 0.001
	Never drinker	119 (19.70)	40 (15.56)	79 (22.77)	
	Moderate alcohol drinker	299 (49.50)	117 (45.53)	182 (52.45)	
	Excessive alcohol drinker	186 (30.79)	100 (38.91)	86 (24.78)	

Smoking, n (%)^b^					< 0.001
	Current smoker	21 (3.48)	20 (7.78)	1 (0.29)	
	Former smoker	69 (11.42)	47 (18.29)	22 (6.34)	
	Never smoker	514 (85.10)	190 (73.93)	324 (93.37)	

BMI (kg/m^2^)^a^;		27.45±6.16	26.69±5.22	28.02±6.72	0.006

BMI, n (%)^b^					0.004
	Underweight	16 (2.65)	4 (1.56)	12 (3.46)	
	Normal weight	222 (36.75)	105 (40.86)	117 (33.72)	
	Overweight	176 (29.14)	85 (33.07)	91 (26.22)	
	Obesity	190 (31.46)	63 (24.51)	127 (36.60)	

Physical activity, n (%)					0.001
	Low	405 (67.05)	154 (59.92)	251 (72.33)	
	Moderate	147 (24.34)	70 (27.24)	77 (22.19)	
	High	41 (6.79)	28 (10.89)	13 (3.75)	
	Very high	11 (1.82)	5 (1.95)	6 (1.73)	

Waist circumference (cm)^a^;		83.68±16.64	85.27±15.15	82.51±17.59	0.039

Abdominal obesity, n (%)^b^					< 0.001
	No	360 (59.60)	202 (78.60)	158 (45.53)	
	Yes	244 (40.40)	55 (21.40)	189 (54.47)	

Diabetes, n (%)^b^		37 (6.13)	16 (6.23)	21 (6.05)	0.929

Hypertension, n (%)^b^		266 (44.04)	119 (46.30)	147 (42.36)	0.334

Hyper-LDL cholesterol, n (%)^b^		138 (22.85)	49 (19.07)	89 (25.65)	0.056

Hyper-total cholesterol, n (%)^b^		163 (26.99)	62 (24.12)	101 (29.11)	0.172

Hypo-total cholesterol, n (%)^b^		291 (48.18)	137 (53.31)	154 (44.38)	0.029

Hypertriglyceridemia, n (%)^b^		326 (53.97)	149 (57.98)	177 (51.01)	0.089

Hypo-HDL cholesterol, n (%)^b^		500 (82.78)	200 (77.82)	300 (86.46)	0.005

Diastolic blood pressure (mmHg)^a^; [min-max]		80.39±12.55 [40-120]	80.95±10.87 [54-112]	79.97±13.67 [40-120]	0.329

Systolic blood pressure (mmHg)^a^; [min-max]		129.20±20.89 [80-195]	131.49±21.11 [100-195]	127.50±20.58 [80-188]	0.020

Glycaemia (mg/dl)^a^; [min-max]		98.90±34.68 [54-448]	97.61±27.70 [55-183]	99.85±39.07 [54-448]	0.411

HDL cholesterol (mg/dl)^a^; [min-max]		33.10±15.27 [3-190]	32.64±13.55 [3-126]	33.44±16.44 [8-190]	0.513

LDL cholesterol (mg/dl)^a^; [min-max]		95.14±74.67 [10-357]	90.58±68.82 [30-357]	98.52±78.67 [10-261]	0.187

Total cholesterol (mg/dl)^a^; [min-max]		159.88±76.23 [37-618]	155.34±68.33 [38-417]	163.24±81.53 [37-618]	0.196

Triglyceride (mg/dl)^a^; [min-max]		168.48±71.81 [24-479]	169.82±70.99 [24-479]	167.48±72.49 [30-430]	0.691

hs-CRP (mg/l)		5.88±16.58 [0.005-192]	3.94±12.28 [0.005-96]	7.32±19.05 [0.005-192]	0.008

^a^Age, BMI, diastolic blood pressure; systolic blood pressure; glycaemia; HDL cholesterol; LDL cholesterol; total cholesterol; and triglyceride were expressed as mean ± SD. BMI, body mass index.

^a^Welch*t*-test; ^b^Chi-square.

MetS, metabolic syndrome; HDL, high-density lipoprotein; LDL, low-density lipoprotein; n, size; min, minimum; max, maximum.

**Table 2:** Features of participants according to metabolic syndrome status in total participants.

		Normal participants	Participants with MetS	*p value* (between normal and MetS)
Total, n (%)		408 (67.55)	196 (32.45)	

Age (years)^a^		41.60±17.61	48.19±15.48	< 0.001

Educational level, n (%)^b^				< 0.001
	Uneducated	6 (1.47)	5 (2.55)	
	Primary	125 (30.64)	75 (38.27)	
	secondary	201 (49.26)	104 (53.06)	
	University	76 (18.63)	12 (6.12)	

Residence area, n (%)^b^				0.648
	Mbouda	292 (71.57)	132 (67.35)	
	Batcham	78 (19.12)	40 (20.41)	
	Galim	22 (5.39)	13 (6.63)	
	Babadjou	16 (3.92)	11 (5.61)	

Drinking, n (%)^b^				0.017
	Never drinker	89 (21.81)	30 (15.31)	
	Moderate alcohol drinker	186 (45.59)	113 (57.65)	
	Excessive alcohol drinker	133 (32.60)	53 (27.04)	

Smoking, n (%)^b^				0.080
	Current smoker	18 (4.41)	3 (1.53)	
	Former smoker	51 (12.50)	18 (9.18)	
	Never smoker	339 (83.09)	175 (89.29)	

BMI (kg/m^2^)^a^		25.48±4.91	31.56±6.46	< 0.001

BMI, n (%)^b^				< 0.001
	Underweight	16 (3.92)	0 (0.00)	
	Normal weight	205 (50.25)	17 (8.67)	
	Overweight	107 (26.23)	69 (35.21)	
	Obesity	80 (19.60)	110 (56.12)	

Physical activity, n (%)				0.007
	Low	257 (62.99)	148 (75.51)	
	Moderate	107 (26.23)	40 (20.41)	
	High	35 (8.58)	6 (3,06)	
	Very high	9 (2.21)	2 (1.02)	

Waist circumference (cm)^a^		77.50±13.89	96.56±14.40	< 0.001

Abdominal obesity, n (%)^b^				< 0.001
	No	351 (86.03)	9 (4.59)	
	Yes	57 (13.97)	187 (95.41)	

Diabetes, n (%)^b^		88 (21.57)	88 (44.90)	< 0.001

Hypertension, n (%)^b^		152 (37.25)	114 (58.16)	< 0.001

Diastolic blood pressure (mmHg)^a^; [min-max]		79.11±12.17 [40-108]	83.04±12.94 [50-120]	< 0.001

Systolic blood pressure (mmHg)^a^; [min-max]		127.32±21.10 [80-195]	133.12±19.92 [80-188]	0.001

Glycaemia (mg/dl)^a^; [min-Max]		93.50±22.38 [55-283]	110.14±49.85 [54-448]	< 0.001

HDL cholesterol (mg/dl)^a^; [min-max]		34.11±14.70 [3-126]	31.00±16.24 [11-190]	0.023

LDL cholesterol (mg/dl)^a^; [min-max]		84.00±65.96 [10-357]	118.26±85.95 [20-175]	< 0.001

Total cholesterol (mg/dl)^a^; [min-max]		152.25±70.01 [37-187]	175.75±85.83 [50-618]	< 0.001

Triglyceride (mg/dl)^a^; [min-max]		163.70±71.72 [24-209]	178.43±71.14 [33-479]	0.018

hs-CRP (mg/l)		2.12±3.94 [0.005-27]	13.72±26.96 [0.005-192]	< 0.001

^a^Age, BMI, diastolic blood pressure; systolic blood pressure; glycaemia; HDL cholesterol; LDL cholesterol; total cholesterol; and triglyceride were expressed as mean ± SD. BMI, body mass index.

^a^Welch*t*-test; ^b^Chi-square.

MetS, metabolic syndrome; HDL, high-density lipoprotein; LDL, low-density lipoprotein; n, size; min, minimum; max, maximum.

**Table 3:** Association between sociodemographic parameters, hs-CRP levels, and metabolic syndrome in total participants.

		Metabolic syndrome		
	Categories/groups	OR	95% CI	p value

Age (years)				
	20 - <30	(ref)		
	30 - <40	4.13	2.22-7.69	<0.001
	40 - <50	4.37	2.53-7.55	<0.001
	50 - <60	5.66	3.17-10.12	<0.001
	60 - <70	2.80	1.50-5.20	0.001
	≥70	3.04	1.51-6.13	0.001

Educational level, n (%)^b^				
	Uneducated	(ref)		
	Primary	0.72	0.21-2.44	0.597
	Secondary	0.62	0.18-2.08	0.440
	University	0.19	0.05-0.72	0.014

Residence area, n (%)^b^				
	Mbouda	(ref)		
	Batcham	1.13	0.73-1.74	0.568
	Galim	1.30	0.63-2.67	0.463
	Babadjou	1.52	0.68-3.37	0.301

Drinking, n (%)^b^				
	Never drinker	(ref)		
	Moderate alcohol drinker	1.80	1.12-2.89	0.015
	Excessive alcohol drinker	1.18	0.70-1.99	0.530

Smoking, n (%)^b^				
	Never smoker	(ref)		
	Former smoker	2.11	0.55-8.01	0.270
	Current smoker	3.09	0.09-10.65	0.073

BMI, n (%)^b^				
	Normal weight	(ref)		
	Underweight	0.00	0.00-0.00	0.971
	Overweight	7.45	4.17-13.30	<0.001
	Obesity	16.34	9.21-28.96	<0.001

Physical activity, n (%)				
	Low	(ref)		
	Moderate	0.65	0.42-0.98	0.041
	High	0.30	0.12-0.72	0.007
	Very high	0.38	0.08-1.81	0.227

hs-CRP (mg/l)	[0-11[	(ref)		
	>11	4.37	2.45-7.77	<0.001

BMI, body mass index; hs-CRP, high sensitivity C-reactive protein; n, size; OR, odds ratio; CI, confidence interval; ref, reference=1.

**Table 4:** Association between the variables related to metabolic syndrome in the total participants.

	Metabolic syndrome		
	OR	95% CI	p value

Abdominal obesity	353.13	136.16-915.81	<0.001
Low HDL	9.28	3.98-21.62	<0.001
High TG	5.62	2.69-11.74	<0.001
High blood pressure	4.43	2.27-8.63	<0.001
Hyperglycemia	4.24	2.11-8.52	<0.001
High total-cholesterol	2.13	1.08-4.21	0.028

HDL, high-density lipoprotein; TG, triglyceride; n, size; OR, odds ratio; CI, confidence interval.

## Data Availability

All data generated or analyzed during this study are included in this published article and the supporting file.

## Abbreviations

ATP III: Adult Treatment Panel III
BMI: Body mass index
BP: Blood pressure
CDC: Centers for Disease Control
CNEC: Cameroon National Ethics Committee
DBP: Diastolic blood pressure
FPG: Fasting plasma glucose
HDL: High-density lipoprotein
hs-CRP: High sensitivity C-reactive protein
HIV: The human immunodeficiency virus
IDF: International Diabetes Federation
LDL: Low-density lipoprotein
MetS: Metabolic syndrome
SBP: Systolic blood pressure
SD: Standard Deviation
TG: Triglyceride.

## Supplementary Materials

Supplementary Materials Supporting file (DOCX): Table S1: variation in the prevalence of metabolic syndrome by sex and age group in the entire population; Table S2: frequency (%) of metabolic syndrome components by sex and age group in the study population; Table S3: frequency (%) of the number of metabolic syndrome components by sex and age group in the study population; Table S4: average frequency of foods intake of participants according to metabolic syndrome; Table S5: associations between the frequency of foods intake and metabolic syndrome in total participants (n = 604).
