# Supplementary material for: Prevalence of Metabolic Syndrome and Its Components in Bamboutos Division's Adults, West Region of Cameroon
Source: Biomed Res Int. 2019 Apr 30;2019:9676984. doi: 10.1155/2019/9676984 (PMC6515192; doi:10.1155/2019/9676984)
Supplement: Supplementary Materials — Supporting file (DOCX): Table S1: variation in the prevalence of metabolic syndrome by sex and age group in the entire population; Table S2: frequency (%) of metabolic syndrome components by sex and age group in the study population; Table S3: frequency (%) of the number of metabolic syndrome components by sex and age group in the study population; Table S4: average frequency of foods intake of participants according to metabolic syndrome; Table S5: associations between the frequency of foods intake and metabolic syndrome in total participants (n = 604). [file 9676984.f1.doc]

**PREVALENCE OF METABOLIC SYNDROME AND ITS COMPONENTS IN MBOUDA ADULTS, WESTERN-CAMEROON**

Wiliane J.T. Marbou^1^, and Victor Kuete^1*^

*^1^Department of Biochemistry, Faculty of Science, University of Dschang, P.O. Box 67 Dschang, Cameroon*

******Corresponding author:***

*Victor Kuete (Prof. Dr.,), Tel : (+237) 677 35 59 27; P.O. Box 67 Dschang, Cameroon; E-mail:* [*kuetevictor@yahoo.fr*](mailto:kuetevictor@yahoo.fr)

***Authors e-mail:***

*Wiliane J.T. Marbou****:***[*marboutakougoum@yahoo.fr*](mailto:marboutakougoum@yahoo.fr)

*Victor Kuete****:***[*kuetevictor@yahoo.fr*](mailto:kuetevictor@yahoo.fr)

**Table S1.** Variation in the prevalence of metabolic syndrome by sex and age group in the entire population.

| Age groups (years) | Participants with MetS | | Male (n=257) | | Female (n=347) | | P-value (between Male and Female) |
| --- | --- | --- | --- | --- | --- | --- | --- |
|  | Size | Prevalence % (95%CI) | Size | Prevalence % (95%CI) | Size | Prevalence % (95%CI) |  |
| 20 - <30 | 27 | 14.44 (9.74-20.31) | 3 | 2.97 (0.62-8.44) | 24 | 27.91 (18.77-38.62) | <0.001 |
| 30 - <40 | 30 | 41.10 (29.71-53.23) | 2 | 6.90 (0.85-22.77) | 28 | 63.64 (47.77-77.59) | <0.001 |
| 40 - <50 | 51 | 42.50 (33.53-51.85) | 14 | 24.56 (14.13-37.76 | 37 | 58.73 (45.62-70.99) | <0.001 |
| 50 - <60 | 44 | 48.89 (38.20-59.65) | 8 | 34.78 (16.38-57.27) | 36 | 53.73 (41.12-66.00) | 0.116 |
| 60 - <70 | 26 | 32.10 (22.15-43.40) | 6 | 26.09 (10.23-48.41) | 20 | 34.48 (22.49-48.12) | 0.465 |
| ≥70 | 18 | 33.96 (21.52-48.27) | 3 | 12.50 (2.66-32.36) | 15 | 51.72 (32.53-70.55) | 0.002 |
| combined age groups | 196 | 32.45 (28.84-36.28) | 36 | 14.01 (10.01-18.86) | 160 | 46.11 (41.94-51.37) | <0.001 |

MetS, Metabolic Syndrome.

**Table S2.** Frequency (%) of metabolic syndrome components by sex and age group in the study population

| **MetS abnormalities** | **Total participants (n= 604)** | **Age groups (years)** | | | | | | **P-value (between age groups**) |
| --- | --- | --- | --- | --- | --- | --- | --- | --- |
|  |  | 20 - <30  (n=187) | 30 - <40  (n=73) | 40 - <50  (n=120) | 50 - <60  (n=90) | 60 - <70  (n=81) | ≥70  (n=53) |  |
| Abdominal obesity, n (%) | 244 (40.40) | 46 (18.85) | 37 (15.16) | 61 (25.00) | 51 (20.90) | 27 (11.07) | 22 (9.02) | <0.001 |
| Low HDL, n (%) | 500 (82.78) | 154 (30.80) | 58 (11.60) | 97 (19.40) | 82 (16.40) | 69 (13.80) | 40 (8.00) | 0.179 |
| High blood pressure, n (%) | 266 (44.04) | 59 (22.18) | 22 (8.27) | 54 (20.30) | 43 (16.17) | 48 (18.05) | 40 (15.04) | <0.001 |
| Hypertriglyceridemia, n (%) | 326 (53.97) | 105 (32.21) | 32 (9.82) | 75 (23.01) | 45 (13.80) | 41 (12.58) | 28 (8.59) | 0.164 |
| Hyperglycemia, n (%) | 37 (6.13) | 0 (00.00) | 6 (16.22) | 9 (24.32) | 7 (18.92) | 8 (21.62) | 7 (18.92) | 0.001 |
|  | | | | | | | | |
|  | **Male (n= 257)** | **Age groups (years)** | | | | | | **P-value (between age groups)** |
| **MetS abnormalities** |  | 20 - <30  (n=187) | 30 - <40  (n=73) | 40 - <50  (n=120) | 50 - <60  (n=90) | 60 - <70  (n=81) | ≥70  (n=53) |  |
| Abdominal obesity, n (%) | 55 (21.40) | 9 (16.36) | 3 (5.45) | 21 (38.18) | 11 (20.00) | 6 (10.91) | 5 (9.09) | <0.001 |
| Low HDL, n (%) | 200 (77.82) | 83 (41.50) | 22 (11.00) | 42 (21.00) | 19 (9.50) | 19 (9.50) | 15 (7.50) | 0.345 |
| High blood pressure, n (%) | 119 (46.30) | 46 (38.66) | 9 (7.56) | 22 (18.49) | 9 (7.56) | 14 (11.76) | 19 (15.97) | 0.004 |
| Hypertriglyceridemia, n (%) | 149 (57.98) | 61 (40.94) | 14 (9.40) | 34 (22.82) | 13 (8.72) | 13 (8.72) | 14 (9.40) | 0.916 |
| Hyperglycemia, n (%) | 16 (6.23) | 0 (00.00) | 3 (18.75) | 3 (18.75) | 2 (12.50) | 4 (25.00) | 4 (25.00) | 0.004 |
|  | | | | | | | | |
|  | **Female (n=347)** | **Age groups (years)** | | | | | | **P-value (between age groups)** |
| **MetS abnormalities** |  | 20 - <30  (n=187) | 30 - <40  (n=73) | 40 - <50  (n=120) | 50 - <60  (n=90) | 60 - <70  (n=81) | ≥70  (n=53) |  |
| Abdominal obesity, n (%) | 189 (54.47) | 37 (19.58) | 34 (17.99) | 40 (21.16) | 40 (21.16) | 21 (11.11) | 17 (8.99) | <0.001 |
| Low HDL, n (%) | 300 (86.46) | 71 (23.67) | 36 (12.00) | 55 (18.33) | 63 (21.00) | 50 (16.67) | 25 (8.33) | 0.386 |
| High blood pressure, n (%) | 147 (42.36) | 13 (8.84) | 13 (8.84) | 32 (21.77) | 34 (23.13) | 34 (23.13) | 21 (14.29) | <0.001 |
| Hypertriglyceridemia, n (%) | 177 (51.01) | 44 (24.86) | 18 (10.17) | 41 (23.16) | 32 (18.08) | 28 (15.82) | 14 (7.91) | 0.197 |
| Hyperglycemia, n (%) | 21 (6.05) | 0 (0.00) | 3 (14.29) | 6 (28.57) | 5 (23.81) | 4 (19.05) | 3 (14.29) | 0.147 |

MetS, Metabolic Syndrome; HDL, High density-lipoprotein; LDL, Low density-lipoprotein; n, size.

**Table S3**. Frequency (%) of the number of metabolic syndrome components by sex and age group in the study population

| **Number of MetS components** | **Total participants (n= 604)** | **Age groups (years)** | | | | | | **P-value (between age groups)** |
| --- | --- | --- | --- | --- | --- | --- | --- | --- |
|  |  | 20 - <30  (n=187) | 30 - <40  (n=73) | 40 - <50  (n=120) | 50 - <60  (n=90) | 60 - <70  (n=81) | ≥70  (n=53) |  |
| 0, n (%) | 20 (3.31) | 8 (40.00) | 6 (30.00) | 2 (10.00) | 2 (10.00) | 2 (10.00) | 0 (0.00) | 0.094 |
| 1 | 75 (12.42) | 37 (49.33) | 10 (13.33) | 16 (21.33) | 3 (4.00) | 8 (10.67) | 1 (1.33) | <0.001 |
| 2 | 181 (29.97) | 73 (40.33) | 21 (11.60) | 29 (16.02) | 19 (10.50) | 25 (13.81) | 14 (7.73) | 0.023 |
| 3 | 207 (34.27) | 55 (26.57) | 18 (8.70) | 39 (18.84) | 39 (18.84) | 31 (14.98) | 25 (12.08) | 0.024 |
| 4 | 86 (14.24) | 13 (15.12) | 15 (17.44) | 23 (26.74) | 18 (20.93) | 11 (12.79) | 6 (6.98) | 0.007 |
| 5 | 36 (5.96) | 1 (2.78) | 2 (5.56) | 13 (36.11) | 8 (22.22) | 5 (13.89) | 7 (19.44) | <0.001 |
|  | | | | | | | | |
| **Number of MetS components** | **Male (n= 257)** | **Age groups (years)** | | | | | | **P-value (between age groups)** |
|  |  | 20 - <30  (n=187) | 30 - <40  (n=73) | 40 - <50  (n=120) | 50 - <60  (n=90) | 60 - <70  (n=81) | ≥70  (n=53) |  |
| 0 | 8 (3.11) | 4 (50.00) | 3 (37.50) | 0 | 0 | 1 (12.50) | 0 | 0.120 |
| 1 | 38 (14.79) | 17 (44.74) | 6 (15.79) | 13 (34.21) | 0 | 1 (2.63) | 1 (2.63) | 0.032 |
| 2 | 92 (35.80) | 40 (43.48) | 13 (14.13) | 17 (18.48) | 10 (10.87) | 6 (6.52) | 6 (6.52) | 0.380 |
| 3 | 80 (31.13) | 35 (43.75) | 4 (5.00) | 16 (20.00) | 5 (6.25) | 7 (8.75) | 13 (16.25) | 0.037 |
| 4 | 26 (10.12) | 5 (19.23) | 2 (7.69) | 8 (30.77) | 5 (19.23) | 5 (19.23) | 1 (3.85) | 0.034 |
| 5 | 10 (3.89) | 0 | 0 | 3 (30.00) | 2 (20.00) | 2 (20.00) | 3 (30.00) | 0.022 |
|  | | | | | | | | |
| **Number of MetS components** | **Female (n=347)** | **Age groups (years)** | | | | | | **P-value (between age groups)** |
|  |  | 20 - <30  (n=187) | 30 - <40  (n=73) | 40 - <50  (n=120) | 50 - <60  (n=90) | 60 - <70  (n=81) | ≥70  (n=53) |  |
| 0 | 12 (3.46) | 4 (33.33) | 3 (25.00) | 2 (16.67 ) | 2 (16.67 ) | 1 (8.33) | 0 | 0.627 |
| 1 | 37 (10.66) | 20 (54.05) | 4 (10.81) | 3 (8.11) | 3 (8.11) | 7 (18.92) | 0 | 0.003 |
| 2 | 89 (25.65) | 33 (37.08) | 8 (8.99) | 12 (13.48) | 9 (10.11) | 19 (21.35) | 8 (8.99) | 0.004 |
| 3 | 127 (36.60) | 20 (15.75) | 14 (11.02) | 23 (18.11) | 34 (18.90) | 24 (18.90) | 12 (9.45) | 0.017 |
| 4 | 60 (17.29) | 8 (13.33) | 13 (21.67) | 15 (25.00) | 13 (21.67) | 6 (10.00) | 5 (8.33) | 0.026 |
| 5 | 26 (7.49) | 1 (3.85) | 2 (7.69) | 10 (38.46) | 6 (23.08) | 3 (11.54) | 4 (15.38) | 0.014 |

P-value (between age groups); MetS, Metabolic syndrome, 0: no metabolic syndrome component; 1: one metabolic syndrome component; 2: two metabolic syndrome component; 3: three metabolic syndrome component; 4: four metabolic syndrome component; 5: five metabolic syndrome component.

**Table S4.** Average frequency of foods intake of participants according to metabolic syndrome.

| **Food (times/month)** | **Total (n = 604)** | **Normal participants (408)** | **Participants with MetS (196)** | **P-value (between normal and MetS participants)** |
| --- | --- | --- | --- | --- |
| Beans mashed potatoes | 5.16 ±6.00 | 5.27 ±6.31 | 4.95 ±5.30 | 0.507 |
| Bread | 10.64 ±10.95 | 10.46 ±10.29 | 11.02 ±12.22 | 0.583 |
| Butter | 1.50 ±4.11 | 1.57 ±4.08 | 1,37 ±4.17 | 0.581 |
| Cabbage, *Solanum nigrum,* | 7.63 ±7.20 | 7.20 ±6.85 | 8.52 ±7.83 | 0.045 |
| Cassava products | 2.37 ±3.93 | 2.48 ±4.09 | 2.12 ±3.58 | 0.280 |
| Corn-Chaff (Corn and Beans) | 0.056 ±0.72 | 0.058 ±0.72 | 0.051 ±0.71 | 0.900 |
| Eggs | 4.33 ±5.62 | 4.47 ±5.78 | 4.04 ±5.27 | 0.361 |
| *Egusi (Mets de pistache*) | 1.82 ±4.62 | 1.64 ±4.38 | 2.21 ±5.09 | 0.179 |
| Fishes | 12.94 ±8.04 | 12.04 ±7.76 | 14.83 ±8.30 | < 0.001 |
| Fruits | 10.67 ±8.62 | 10.79 ±8.43 | 10.42 ±9.03 | 0.634 |
| Fufu corn (*couscous* maïs) | 6.81 ±5.90 | 6.37 ±5.63 | 7.74 ±6.34 | 0.010 |
| Groundnut | 5.73 ±6.80 | 5.72 ±6.82 | 5.74 ±6.77 | 0.953 |
| Koki | 0.045 ±0.57 | 0.032 ±0.36 | 0.071 ±0.86 | 0.538 |
| Meat (pork, mutton, goat) | 6.75 ±7.04 | 6.69 ±6.84 | 6.88 ±7.45 | 0.758 |
| Milk, Yogurt | 4.66 ±7.34 | 4.71 ±7.29 | 4.56 ±7.47 | 0.822 |
| Palm Oil | 14.93 ± 10.10 | 14.78 ±10.16 | 15.23 ±9.99 | 0.608 |
| Pasta | 3.80 ±5.07 | 4.47 ±5.56 | 2.40 ±3.48 | < 0.001 |
| Potatoes | 0.14 ±1.30 | 0.19 ±1.49 | 0.05 ±0.71 | 0.133 |
| Refined oil | 14.81 ±10.06 | 15.14 ±10.12 | 14.11 ±9.93 | 0.234 |
| Rice | 9.55 ±6.95 | 9.91 ±7.03 | 8.80 ±6.74 | 0.061 |
| Sugar products | 2.98 ±6.22 | 3.36 ±6.41 | 2.21 ±5.75 | 0.027 |
| *Taro (*mashed/pounded achu coco) | 4.32 ±4.75 | 4.12 ±4.39 | 4.75 ±5.42 | 0.158 |
| Topsi Banana (Banane Malaxée) | 0.19 ±1.83 | 0.14 ±1.44 | 0.29 ±2.46 | 0.411 |

mean±SD

**Table S5**. Associations between the frequency of foods intake and metabolic syndrome in total participants (n = 604).

|  | **Times/month: OR (95%-CI)** | | | | **P-trend**^a^ |
| --- | --- | --- | --- | --- | --- |
|  | [0-4[ | [4-13[ | [13-25[ | [25-90] |  |
| Beans mashed potatoes | (ref) | 1.30 (0.85-1.99) | 0.54 (.13-2.20) | 1593867.13  (0.00- >1.0E12) | 0,238 |
| Bread | (ref) | 0.89 (0.54-1.48) | 1.37 (0.66-2.84) | 1.38 (0.67-2.85) | 0,336 |
| Butter | (ref) | 1.38 (0.69-2.76) | 1.06 (0.17-6.70) | 5.98 (0.18-191.39) | 0.276 |
| Cabbage, *Solanum nigrum,* | (ref) | 1.00 (0.63-1.60) | 1.53 (0.69-3.37) | 2.81 (0.19-41.38) | 0,277 |
| Cassava products | (ref) | 0.70 (0.44-1.12) | 0.00  (0.00- >1.0E12) | NDIF | 0.283 |
| Corn-Chaff (Corn and Beans) | (ref) | 2.39 (0.12-46.30) | NDIF | NDIF | 0.221 |
| Eggs | (ref) | 0.67 (0.41-1.07) | 1.11 (0.61-2.01) | 0.64 (0.04-8.72) | 0.299 |
| *Egusi (Mets de pistache*) | (ref) | 1.15 (0.65-2.01) | 3.54 (0.85-14.77) | NDIF | 0,266 |
| Fishs | (ref) | 0.62 (0.28-0.97) | 0.12 (0.001-0.27) | 0.00 (0.00-0.00) | 0.035 |
| Fruits | (ref) | 0.68 (0.39-1.19) | 0.69 (0.33-1.42) | 1.60 (0.39-6.62) | 0,269 |
| Fufu corn (*couscous* maïs) | (ref) | 0.85 (0.53-1.38) | 1.46 (0.64-3.32) | 6.09 (0.31-119.88) | 0.348 |
| Groundnut | (ref) | 1.77 (0.98-3.21) | 1.39 (0.87-2.22) | NDIF | 0,231 |
| Koki | (ref) | 6.01 (0.46-77.65) | NDIF | NDIF | 0,285 |
| Meat (pork, mutton, goat) | (ref) | 0.68 (0.43-1.06) | NDIF | 1.39 (0.61-3.18) | 0.339 |
| Milk, Yogurt | (ref) | 1.20 (0.75-1.92) | 1.28 (0.51-3.23) | 1.16 (0.27-4.99) | 0,266 |
| Palm Oil | (ref) | 0.86 (0.45-1.65) | 0.72 (0.36-1.47) | 1.66 (0.70-3.94) | 0,186 |
| Pasta | (ref) | 0.55 (0.36-0.86) | 0.24 (0.05-1.21) | NDIF | 0.237 |
| Potatoes | (ref) | 0.73 (0.04-12.81( | 0.00 (0.00- >1.0E12) | NDIF | 0.266 |
| Refined oil | (ref) | 1.01 (0.56-1.84) | 0.70 (0.37-1.30) | 0.99 (0.44-2.22) | 0.254 |
| Rice | (ref) | 0.89 (0.50-1.60) | 0.88 (0.39-2.02) | 0.00  (0.00- >1.0E12) | 0,302 |
| Sugar product | (ref) | 0.69 (0.34-1.43) | 0.99 (0.49-1.99) | 6.12 (0.12-311.78) | 0.341 |
| *Taro (*mashed/pounded achu coco) | (ref) | 0.69 (0.44-1.08) | 1.94 (0.47-7.97) | 1618379.34 (0.00- >1.0E12) | 0,281 |
| Topsi Banana (*Banane Malaxée*) | (ref) | 1.59 (0.05-50.54) | 16.91 (0.91-313.06) | NDIF | 0.268 |

^a^: Adjusted for the age, residence, Educational level, smoking status, drinking status, physical activity level, and BMI.

NDIF; No dietary intake frequency.
